# Type III Transforming Growth Factor-*β* Receptor RNA Interference Enhances Transforming Growth Factor *β*3-Induced Chondrogenesis Signaling in Human Mesenchymal Stem Cells

**DOI:** 10.1155/2018/4180857

**Published:** 2018-08-08

**Authors:** Shuhui Zheng, Hang Zhou, Zhuohui Chen, Yongyong Li, Taifeng Zhou, Chengjie Lian, Bo Gao, Peiqiang Su, Caixia Xu

**Affiliations:** ^1^Research Center for Translational Medicine, The First Affiliated Hospital of Sun Yat-sen University, Guangzhou, China; ^2^Department of Orthopedic Surgery, The First Affiliated Hospital of Sun Yat-sen University, Guangzhou, China; ^3^Oral and Maxillofacial Surgery, Guanghua School of Stomatology, Hospital of Stomatology, Sun Yat-sen University, Guangzhou, China; ^4^Department of Spine Surgery, Sun Yat-Sen Memorial Hospital, Sun Yat-sen University, Guangzhou, China

## Abstract

The type III transforming growth factor-*β* (TGF-*β*) receptor (T*β*RIII), a coreceptor of the TGF-*β* superfamily, is known to bind TGF-*β*s and regulate TGF-*β* signaling. However, the regulatory roles of T*β*RIII in TGF-*β*-induced mesenchymal stem cell (MSC) chondrogenesis have not been explored. The present study examined the effect of T*β*RIII RNA interference (RNAi) on TGF-*β*3-induced human MSC (hMSC) chondrogenesis and possible signal mechanisms. A lentiviral expression vector containing T*β*RIII small interfering RNA (siRNA) (SiT*β*RIII) or a control siRNA (SiNC) gene was constructed and infected into hMSCs. The cells were cultured in chondrogenic medium containing TGF-*β*3 or control medium. T*β*RIII RNAi significantly enhanced TGF-*β*3-induced chondrogenic differentiation of hMSCs, the ratio of type II (T*β*RII) to type I (T*β*RI) TGF-*β* receptors, and phosphorylation levels of Smad2/3 as compared with cells infected with SiNC. An inhibitor of the TGF-*β* signal, SB431542, not only inhibited T*β*RIII RNAi-stimulated TGF-*β*3-mediated Smad2/3 phosphorylation but also inhibited the effects of T*β*RIII RNAi on TGF-*β*3-induced chondrogenic differentiation. These results demonstrate that T*β*RIII RNAi enhances TGF-*β*3-induced chondrogenic differentiation in hMSCs by activating TGF-*β*/Smad2/3 signaling. The finding points to the possibility of modifying MSCs by T*β*RIII knockdown as a potent future strategy for cell-based cartilage tissue engineering.

## 1. Introduction

Cell-based cartilage tissue engineering provides a feasible way of regenerating damaged cartilage tissue caused by trauma or joint diseases. Mesenchymal stem cells (MSCs), common precursor cells of chondrocytes, are the basis for the development of cartilage and represent promising cells for use in stem cell therapy [1, 2]. However, cartilage tissue formed by MSC-derived chondrocytes is not the same as that of native articular cartilage and has poor functional properties [3, 4]. Understanding the molecular mechanisms that control chondrogenic differentiation of MSCs and enhancing chondrogenic activities of cells are crucial to improve cartilage regeneration by MSCs.

Chondrogenic differentiation is potently induced by growth factors [2, 5]. Transforming growth factor-*β*3 (TGF-*β*3), a member of the transforming growth factor-*β* (TGF-*β*) superfamily, induces chondrogenic differentiation of MSCs [6]. TGF-*β* signaling is initiated by the binding of TGF-*β* to type II TGF-*β* receptors (T*β*RII) and then forms heteromeric complexes with type I receptors (T*β*RI) [7]. These complexes further phosphorylate cytoplasmic effector molecules Smad2 and Smad3, which are translocated to the nucleus, where they modulate the expression of target genes, such as SOX9 and collagen type II (COL II) [8].

In addition to kinase receptors, the type III TGF-*β* receptor (T*β*RIII), also known as betaglycan, participates in ligand binding of the TGF-*β* superfamily and signaling [9, 10]. T*β*RIII is a membrane-anchored proteoglycan found in many cell types. The main function of this receptor was thought to be binding members of the TGF-*β* family, including TGF-*β* isoforms TGF-*β*1–TGF-*β*3, and presenting them to type II receptors [11, 12]. However, according to the recent literature, T*β*RIII seems to play complex roles in cellular processes. For example, studies reported that in some cell lines, T*β*RIII significantly enhanced the response to TGF-*β* by increasing the affinity of T*β*RII for TGF-*β* [12, 13]. Studies also showed that T*β*RIII functioned as a potent inhibitor of TGF-*β* signaling in other types of cells, such as renal epithelial cells [14, 15]. Our previous study demonstrated that human MSCs (hMSCs) expressed abundant T*β*RIII and that TGF-*β*3 stimulation clearly repressed T*β*RIII expression in MSCs and induced MSC chondrogenic differentiation [16]. These results suggested that the effect of TGF-*β*3 on MSC chondrogenesis might be associated with low expression of T*β*RIII. However, the role of T*β*RIII in TGF-*β*-induced chondrogenic differentiation of MSCs is unknown.

In the present study, we studied the biological effects of T*β*RIII on TGF-*β*3-induced MSC chondrogenesis. We demonstrated that T*β*RIII RNA interference (RNAi) enhanced TGF-*β*-induced chondrogenic differentiation of hMSCs by activating TGF-*β* and Smad2/3 signaling.

## 2. Materials and Methods

### 2.1. Isolation and Culture of hMSCs

The study was approved by the ethics committee of the First Affiliated Hospital of Sun Yat-sen University in accordance with the Declaration of Helsinki, and all the subjects provided written informed consent. hMSCs were isolated and purified from bone marrow samples of 3 healthy volunteer donors by density gradient centrifugation, as described previously [17]. Briefly, bone marrow samples (8–10 ml) were diluted with 10 ml phosphate-buffered saline (PBS). Cells were then fractionated using a Lymphoprep (MP Biomedicals LLC., Santa Ana, CA, USA) density gradient by centrifugation at 500 ×g for 20 minutes. Interfacial mononuclears were collected and cultured in low-glucose Dulbecco's modified Eagle medium (L-DMEM) (Gibco; Invitrogen Corporation, NY, USA), supplemented with 10% fetal bovine serum (FBS) (Gibco; Invitrogen Corporation, NY, USA) under 37°C and 5% CO_2_. Cells were passaged when they reached approximately 80% confluence. Passages 3 to 5 cells were used for the experimental protocols.

### 2.2. T*β*RIII Small Interfering RNA (siRNA) Design and Lentiviral Vector Construction

The human T*β*RIII cDNA sequence (GenBank accession number: NM_003243) was searched for siRNA target sequences. Four target sequences were selected, AAGCATGAAGGAACCAAAT, TGCTTTATCTCTCCATATT, ACCTGAAATCGTGGTGTTT, and AGTTGGTAAAGGGTTAATA. A scrambled sequence, TTCTCCGAACGTGTCACGT, was used as a negative control (NC). DNA oligos containing the target sequence were synthesized, annealed, and inserted into a green fluorescent protein (GFP) lentiviral expression vector GV248 (GeneChem, Shanghai, China). The ligated production was transformed into *Escherichia coli* DH5*α* cells. Briefly, 100 *μ*l of DH5*α* cells was mixed with 2 *μ*l ligated product at 42°C for 90 s. The mixtures were added on Luria-Bertani (LB) media (ATCC, USA) containing ampilillin (50 *μ*g/ml) and incubated at 37°C for 16 h. The transformant was identified by polymerase chain reaction (PCR) and DNA sequencing.

### 2.3. Lentiviral Production and Infection

A lentivirus T*β*RIII (LV-T*β*RIII) siRNA-mix and lentivirus normal control (LV-NC) virus were produced by plasmid cotransfection of 293T cells. Briefly, 293T cells were transfected with DNA mix (pGC-LV vector, 20 *μ*g; pHelper 1.0 vector, 15 *μ*g; and pHelper 2.0 vector, 10 *μ*g) (GeneChem, Shanghai, China) and 100 *μ*l of Lipofectamine 2000 reagent (Invitrogen, NY, USA), according to the manufacturer's instructions. The viral supernatant was harvested 48 hours (h) after transfection, and the concentrated viral titer was determined. The viral supernatant was added into target MSCs at multiplicity of infection (MOI 10). Before infection, 5 × 10^4^/ml of MSCs were seeded onto a 60 cm^2^ cell culture dish overnight. The cells were then infected with 100 *μ*l of 1 × 10^8^ TU/ml virus and 5 *μ*g/ml of polybrene, following the manufacturer's instructions. Then, 10 h after infection, the cells were incubated with L-DMEM containing 10% FBS. Three days after infection, GFP expression in cells was observed by a fluorescence microscope (Olympus, Japan). T*β*RIII expression was detected by the real-time polymerase chain reaction (PCR) and Western blot analysis.

### 2.4. Chondrogenic Differentiation of hMSCs in Micromass Culture

Chondrogenic differentiation of hMSCs was performed using a modified micromass culture system according to a previously described method [18]. Briefly, MSCs being infected with T*β*RIII siRNA (SiT*β*RIII) or control siRNA (SiNC), or without infection, were harvested and resuspended at 2 × 10^7^ cells/ml. Cell droplets (4 × 10^5^/20 *μ*l) were placed carefully in each well of 24-well plates for 2 h, followed by the addition of control medium or chondrogenic medium at 37°C/5% CO_2_. The control medium consisted of high-glucose DMEM (H-DMEM), supplemented with 50 *μ*g/ml of vitamin C, 100 nM dexamethasone, 1 mM sodium pyruvate, 40 *μ*g/ml of proline, and ITS+Universal Culture Supplement Premix (BD Biosciences, NY, USA). The chondrogenic medium consisted of the control medium, added by 10 ng/ml of TGF-*β*3 (PeproTech, Rocky Hill, USA). After 24 h, the cell droplets became spherical. The medium was changed every 3 days.

Cell pellets from uninfected MSCs were divided into a control group and TGF-*β*3 group according to the control medium and chondrogenic medium and used for T*β*RIII analysis. The cells were harvested on day 7 after chondrogenic induction. The pellets from infected MSCs were divided into the following four groups for identification of chondrogenic differentiation and TGF-*β*/Smad signaling: C-SiNC group (SiNC-infected cells cultured in control medium), C-SiT*β*RIII group (SiT*β*RIII-infected cells cultured in control medium), T-SiNC group (SiNC-infected cells cultured in chondrogenic medium), and T-SiT*β*RIII group (SiT*β*RIII-infected cells cultured in chondrogenic medium). The cells were harvested on day 14 for identification of chondrogenic differentiation, and on day 7 for T*β*RI and T*β*RII analysis. All experiments were performed by using 3 biological replicate samples each group.

### 2.5. RNA Extraction and Real-Time PCR Analysis

Total RNA was isolated from transfected MSCs or pellets using an RNAsimple Total RNA Kit (Tiangen, Beijing, China). Total RNA was then converted into cDNA using a PrimeScript® RT Reagent Kit (Takara, Osaka, Japan) according to the manufacturer's instructions. Real-Time PCR assay was performed in triplicate in a Real-Time PCR system (Bio-Rad Laboratories, Hercules, CA, USA) by using SYBR Green I Master Mix (Takara, Osaka, Japan). The following genes were examined: COL II, alpha 1 (COL2A1), SRY- (sex determining region Y-) box 9 [SOX9], T*β*RI, T*β*RII, and T*β*RIII. The primer sequences are listed in Table 1. The relative expression levels for each target gene were calculated by referencing to the internal controls glyceraldehyde-3-phosphate dehydrogenase (GAPDH) and *β*-actin using the 2^–ΔΔCT^ method.

### 2.6. Histology and Immunohistochemistry

The pellets were fixed in 4% paraformaldehyde and embedded in paraffin. 4 *μ*m sections were deparaffinized, rehydrated through decreasing concentrations of ethanol, and stained with 0.1% Alcian blue (Sigma-Aldrich, St. Louis, USA) for glycosaminoglycan (GAG) analysis. For immunohistochemistry analysis, the sections were blocked with 1/100 diluted goat serum for 15 min and then reacted overnight at 4°C with the appropriate primary antibody against human COL II polyclonal antibodies (Abzoom Biolabs, Dallas, TX, USA) and human T*β*RIII antibody (Santa Cruz, Dallas, USA) followed by biotinylated goat anti-rabbit immunoglobulin G (IgG) (EarthOx, SFO, USA) for 30 min. The sections were incubated with peroxide-conjugated streptavidin and stained with 3,3′-diaminobenzidine tetrahydrochloride (DAB) (Jinshan Jinqiao, Beijing, China).

For fluorescent immunohistochemistry, the pellets from transfected MSCs were frozen and then sectioned using a Leica CM1950 microtome (Leica, Germany) on day 7 after chondrogenic induction. Tissue sections that were 4 *μ*m thick were permeabilized with 0.1% Triton X for 5 min and blocked with 5% BSA for 1 h. The sections were then incubated with primary antibodies against T*β*RI (Santa Cruz, Dallas, USA) and human T*β*RII antibody (RD Systems, USA), diluted at 1 : 50 at 4°C overnight. FITC-conjugated secondary antibody (K00018968; Dako North America Inc., Dako, Denmark) diluted at 1 : 100 was applied for 1 h. The sections were then stained with 4′-6-diamidino-2-phenylindole (1 mg/ml) and visualized using a Zeiss LSM 710 confocal microscope (Carl Zeiss, Heidelberg, Germany).

### 2.7. Glycosaminoglycan (GAG) Quantitation

The pellets were harvested on day 14 and papain-digested for 16 h at 65°C. An aliquot of 40 *μ*l lysate was reacted with 1,9-dimethyl-methylene blue (DMMB) (Sigma-Aldrich, St. Louis, USA) for GAG analysis. The absorbance at 525 nm was measured using an Automatic Microplate Reader (BioTek, Winooski, Vermont, USA). Total GAG was calculated by a standard curve obtained with shark chondroitin sulfate (Sigma-Aldrich, MO, USA). The total amount of DNA was quantified by reacting with 0.7 *μ*g/ml Hoechst 33258 solution (Sigma-Aldrich, St. Louis, USA). The reaction product was measured using a Synergy Microplate Reader (BioTek, Winooski, Vermont, USA). The results of GAG quantification were normalized to the DNA content.

### 2.8. Western Blot

For T*β*RIII RNAi identification, proteins were extracted from MSCs 72 h after transfection. For detection of Smad2/3 phosphorylation, proteins were extracted from pellets after 24 h of chondrogenic induction. The protein concentration was quantified by a BCA Protein Assay Kit (CWBio, Beijing, China). 100 *μ*g proteins was subjected to 6% SDS-PAGE and electrotransferred onto PVDF membrane (Millipore, Boston, USA) at 250 mV for 100 min. After blocking with 5% skim milk and Tris-buffered saline containing 0.1% Tween-20, the PVDF membranes were incubated with antibodies against human T*β*RIII antibody (Santa Cruz, Dallas, USA), rabbit anti-phospho-Smad2 (Ser465/467)/Smad3 (Ser423/425) (Cell Signaling, Danvers, USA), rabbit anti-Smad2/3 (Cell Signaling, Danvers, USA), and anti-GAPDH monoclonal antibody (EarthOx, SFO, USA) followed by incubation with HRP-conjugated corresponding secondary antibodies. The signals were detected using SuperSignal West Pico Chemiluminescent Substrate (Pierce, NY, USA). Protein levels in phosphorylated-Smad2/3 (P-Smad2/3) were normalized to those of total Smad2/3 quantities or GAPDH.

### 2.9. Inhibition of TGF-*β*/Smad Signaling

The infected cells were treated with or without SB431542 (Sigma-Aldrich, St. Louis, USA), a selective inhibitor of activin receptor-like kinase 5 (ALK5) (T*β*RI) [19], for 2 h before being cultured in chondrogenic medium or control medium. After 24 h of chondrogenic induction, the cells were collected, and the expression of P-Smad2/3 and that of Smad2/3 was detected by a Western blot. After 14 days of chondrogenic induction, the chondrogenic differentiation ability of hMSCs was assayed by detecting protein and gene expression.

### 2.10. Statistical Analysis

All quantitative data were presented as mean values ± standard errors (S.E.). All the statistical analysis was performed using SPSS 16.0 statistical software (SPSS, Chicago, IL, USA). For comparisons of two groups, independent student's *t-*test was performed; for comparisons of multiple groups, one-way ANOVA followed by an LSD *t*-test was performed. *P* < 0.05 was chosen as the threshold of significance.

## 3. Results

### 3.1. TGF-*β*3 Inhibited the Expression of T*β*RIII in hMSCs

**Figure 1:**
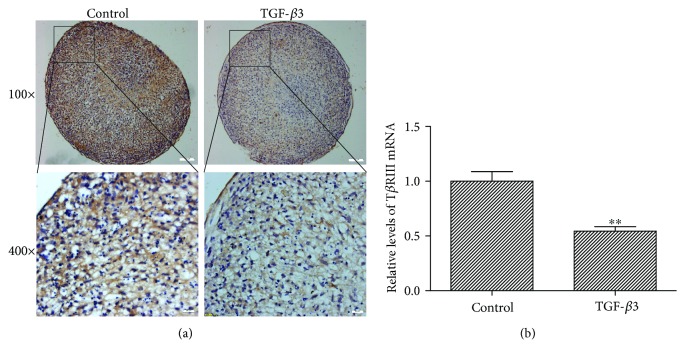
TGF-*β*3 inhibited the expression of T*β*RIII during chondrogenic differentiation of hMSCs. (a) hMSCs were cultured in chondrogenic medium including TGF-*β*3 or control medium without TGF-*β*3 for 7 days and subjected to immunohistochemistry analysis for T*β*RIII (upper panel, scale bar = 100 *μ*m; lower panel, scale bar = 20 *μ*m; the T*β*RIII is stained in brown, cell nucleus is stained in blue). (b) qPCR analysis of T*β*RIII mRNA level in hMSCs cultured in chondrogenic medium or control medium for 7 days. The T*β*RIII mRNA level was lower in hMSCs cultured in chondrogenic medium containing TGF-*β*3 as compared with that of cells cultured in control medium. Error bars represent the means ± SD, *n* = 3. ^∗∗^*P* < 0.01 versus control.

To ascertain whether hMSCs express T*β*RIII and TGF-*β*3 could regulate T*β*RIII expression level, we detected the expression of T*β*RIII during TGF-*β*3-induced hMSC chondrogenesis by immunohistochemistry staining and quantitative PCR. The results showed that hMSCs expressed abundant T*β*RIII protein (Figure 1(a), left panels) and high T*β*RIII mRNA (*P* < 0.01, Figure 1(b)). Exogenous TGF-*β*3 clearly reduced T*β*RIII expression in hMSCs at protein and mRNA levels (Figures 1(a) and 1(b)).

### 3.2. Viral Infection and Suppression of T*β*RIII Expression at mRNA and Protein Levels

**Figure 2:**
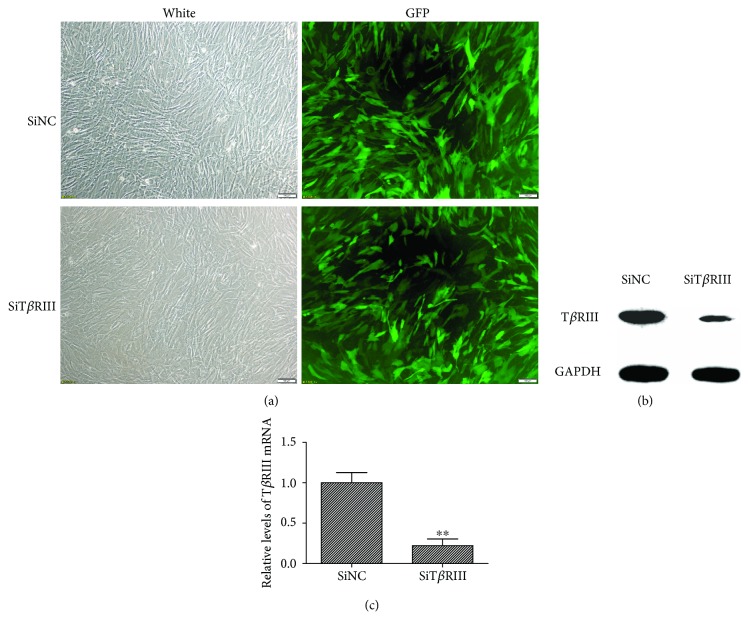
Identification and efficiency of lentiviral infection. (a) GFP fluorescence imaging confirmed that the majority of hMSCs were GFP positive 72 h after they were infected by T*β*RIII siRNA (SiT*β*RIII) or SiNC virus. Scale bar = 100 *μ*m. (b) Western blot showed that T*β*RIII siRNA clearly inhibited the expression of the T*β*RIII protein. (c) qPCR confirmed that the expression profiles of T*β*RIII mRNA decreased significantly in the SiT*β*RIII groups as compared with those in the SiNC group. Error bars represent the means ± SD, *n* = 3. ^∗∗^*P* < 0.01 versus SiNC group.

In order to investigate the role of T*β*RIII in hMSC chondrogenesis, we infected hMSCs with LV-T*β*RIII siRNA-mix and identified the silencing effect on T*β*RIII. Following viral infection for 72 h, most of the cells exhibited high GFP expression under fluorescence microscopy (Figure 2(a)). As compared with the control group, the expression profile of T*β*RIII mRNA and that of the T*β*RIII protein decreased significantly in the SiT*β*RIII groups, with mRNA expression decreased by 77.65% (Figures 2(b) and 2(c)).

### 3.3. T*β*RIII RNAi Enhanced TGF-*β*3-Induced Chondrogenic Differentiation of hMSCs

**Figure 3:**
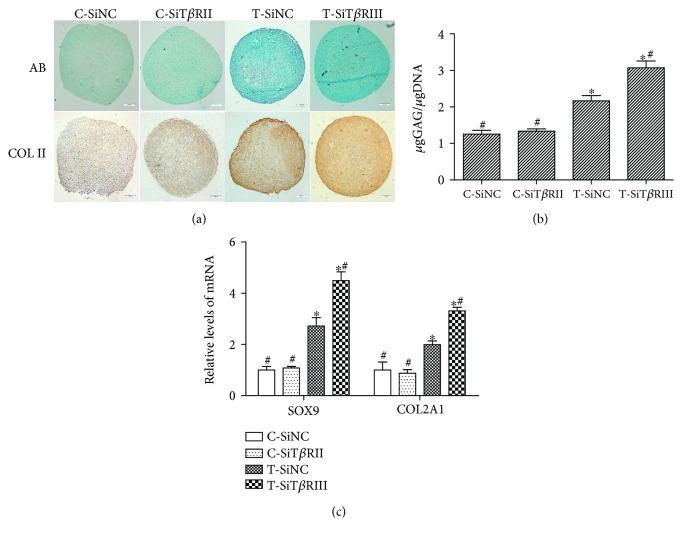
T*β*RIII RNAi enhanced TGF-*β*3-induced chondrogenic differentiation of hMSCs. SiNC- or SiT*β*RIII-infected cells were cultured in control medium (C-SiNC group; C-SiT*β*RIII group) or chondrogenic medium containing TGF-*β*3 (T-SiNC group; T-SiT*β*RIII group) for 14 days. (a) Upper panels show glycosaminoglycan (GAG) expression by Alcian blue staining; lower panels show COL II expression by immunohistochemistry. Scale bar = 100 *μ*m. (b) GAG content was quantitatively analyzed and normalized by DNA content. (c) Real-time PCR analysis of COL2A1 and SOX9 mRNA levels in hMSCs from different groups. Error bars represent the means ± SD, *n* = 3. ^∗^*P* < 0.05 versus C-SiNC group; ^#^*P* < 0.05 versus T-SiNC group.

We then investigated the effects of T*β*RIII RNAi on the TGF-*β*3-induced chondrogenic differentiation of hMSCs. As shown in Figure 3, T*β*RIII RNAi had no obvious effects on GAG and COL II secretion (Figures 3(a) and 3(b); *P* > 0.05) or any noticeable effects on the expression of cartilage-specific genes (SOX9 and COL2A1) when hMSCs were cultured in control medium (Figure 3(c); *P* > 0.05). However, T*β*RIII RNAi significantly enhanced TGF-*β*3-induced chondrogenic differentiation of hMSCs as compared with that of cells infected with SiNC (Figures 3(a)–3(c); *P* < 0.05).

### 3.4. T*β*RIII RNAi Increased the Ratio of T*β*RII to T*β*RI

**Figure 4:**
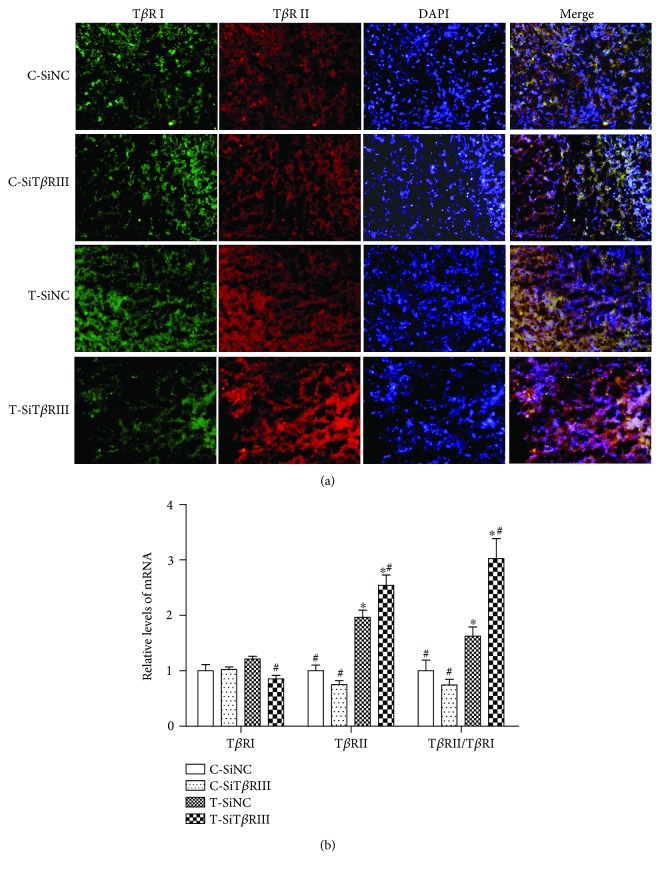
T*β*RIII RNAi increased the ratio of T*β*RII to T*β*RI. SiNC- or SiT*β*RIII-infected cells were cultured in control medium (C-SiNC group; C-SiT*β*RIII group) or chondrogenic medium containing TGF-*β*3 (T-SiNC group; T-SiT*β*RIII group) for 7days (*n* = 3). (a) The expression of T*β*RI and that of T*β*RII were visualized by immunofluorescence staining using anti-T*β*RI (green) and anti-T*β*RII (red) antibodies. Nuclei were counterstained using DAPI (blue). The far right panels show merged images. Scale bar = 20 *μ*m. (b) mRNA expression of T*β*RI and T*β*RII and the ratio of T*β*RII to T*β*RI by real-time RT-PCR. ^∗^*P* < 0.05 versus C-SiNC group; ^#^*P* < 0.05 versus T-SiNC group.

To explore the mechanism on T*β*RIII RNAi regulating TGF-*β*3-induced chondrogenic differentiation of hMSCs, we further analyzed the effect of T*β*RIII RNAi on T*β*RI and T*β*RII expression and downstream Smad2/3 signaling during TGF-*β*3-induced chondrogenesis. Analysis of T*β*RI and T*β*RII expression revealed that both T*β*RI mRNA levels and protein expression had no difference between the cells in the C-SiNC, C-SiT*β*RIII, and T-SiNC groups (Figures 4(a) and 4(b); *P* > 0.05). However, T*β*RI mRNA and protein expression levels were decreased in the T-SiT*β*RIII group as compared with those in the other groups (Figures 4(a) and 4(b); *P* < 0.05). Neither the expression of the T*β*RII gene (Figure 4(a)) nor that of the protein (Figure 4(b)) was increased in the C-SiT*β*RIII group as compared with that in the C-SiNC group (*P* > 0.05). However, as compared with the C-SiNC group, both the T-SiNC and T-SiT*β*RIII groups had enhanced mRNA levels of T*β*RII (Figure 4(a); *P* < 0.05) and T*β*RII expression (Figure 4(b)). The expression of the T*β*RII gene, as well as that of the T*β*RII protein, was higher in the T-SiT*β*RIII group as compared with that in the T-SiNC group. The ratio of T*β*RII to T*β*RI in the T-SiNC and T-SiT*β*RIII groups was higher than that in the C-SiNC and C-SiT*β*RIII groups (Figure 4(a); *P* < 0.05 and *P* < 0.01, resp.). T*β*RII/T*β*RI levels increased dramatically in the T-SiT*β*RIII groups as compared with those in other groups (Figure 4(a); *P* < 0.05).

### 3.5. T*β*RIII RNAi Strengthened TGF-*β*3-Mediated Phosphorylation of Smad2/3

**Figure 5:**
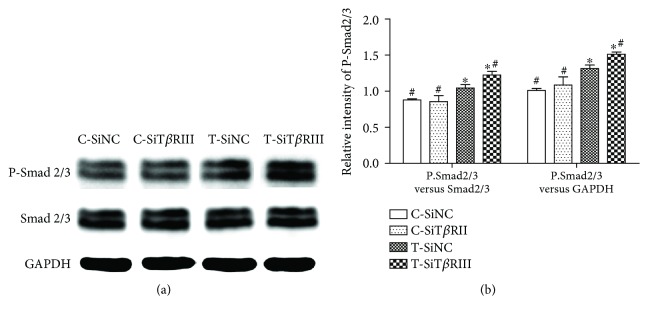
T*β*RIII RNAi strengthened TGF-*β*3-mediated Smad2/3 phosphorylation. SiNC- or SiT*β*RIII-infected cells were cultured in control medium (C-SiNC group; C-SiT*β*RIII group) or chondrogenic medium containing TGF-*β*3 (T-SiNC group; T-SiT*β*RIII group) and harvested after 24 h. (a) A Western blot of protein levels of P-Smad2/3, total Smad2/3, and GAPDH. (b) Quantification of protein levels of P-Smad2/3 normalized to total levels of Smad2/3 or GAPDH. Error bars represent the means ± SD, *n* = 3. ^∗^*P* < 0.05 versus C-SiNC group; ^#^*P* < 0.05 versus T-SiNC group.

Analysis of phosphorylation of Smad2/3 revealed that T*β*RIII-RNAi did not affect the expression of P-Smad2/3 in control medium. However, phosphorylation of Smad2/3 was obviously activated in T-SiNC and T-SiT*β*RIII groups. Interestingly, when cells were infected with SiT*β*RIII, the activation of P-Smad2/3 was further enhanced (Figures 5(a) and 5(b)).

### 3.6. SB431542 Blocked T*β*RIII RNAi-Activated TGF-*β*3-Mediated Phosphorylation of Smad2/3

**Figure 6:**
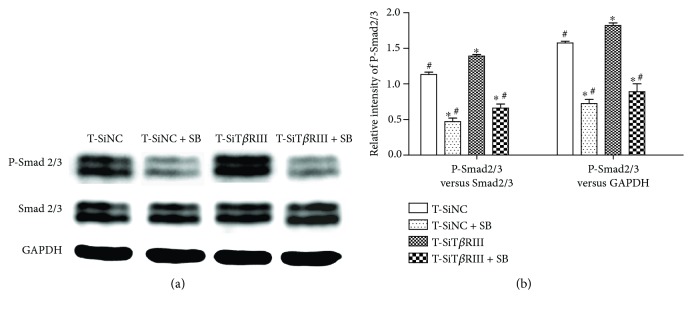
SB431542 blocked T*β*RIII RNAi-activated TGF-*β*3-mediated phosphorylation of Smad2/3. SiNC- or SiT*β*RIII-infected hMSCs were cultured in chondrogenic medium, supplemented with TGF-*β*3 (T-SiNC group or T-SiT*β*RIII group) and exposed to SB431542 treatment for 2 h before treatment with TGF-*β*3 (T-SiNC + SB group or T-SiT*β*RIII + SB group). Samples were harvested after 24 h. (a) Western blot of protein levels of P-Smad2/3, total Smad2/3, and GAPDH. (b) Quantification of protein levels of P-Smad2/3 normalized to total levels of Smad2/3 and GAPDH. Error bars represent the means ± SD, *n* = 3. ^∗^*P* < 0.05 versus T-SiNC group, ^#^*P* < 0.05 versus T-SiT*β*RIII group.

SB431542 was identified as a specific inhibitor of T*β*RI and TGF-*β* signaling [19]. Therefore, we tested whether SB431542 blocked T*β*RIII RNAi-activated TGF-*β* signaling. The results showed that SB431542 inhibited both TGF-*β*3-activated Smad2/3 phosphorylation and T*β*RIII RNAi-activated TGF-*β*3-mediated Smad2/3 phosphorylation (Figure 6(a)). The results of the statistical analysis revealed decreased ratios of P-Smad2/3 to Smad2/3 and P-Smad2/3 to GAPDH in the T-SiNC + SB and T-SiT*β*RIII + SB groups as compared with those in the T-SiNC and T-SiT*β*RIII groups (*P* < 0.05), as shown in Figure 6(b). There was no statistical difference in P-Smad2/3 levels in the T-SiNC + SB group versus those in the T-SiT*β*RIII + SB group (*P* > 0.05) (Figure 6(b)). These data showed that SB431542 completely inhibited TGF-*β*3-mediated Smad2/3 phosphorylation, activated by T*β*RIII RNAi.

### 3.7. SB431542 Inhibited T*β*RIII RNAi-Enhanced TGF-*β*3-Induced Chondrogenic Differentiation of hMSCs

**Figure 7:**
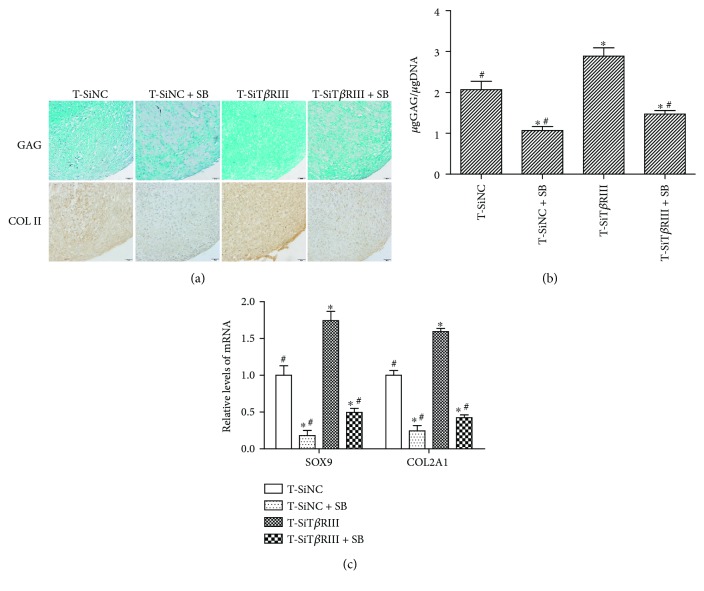
SB431542 inhibited T*β*RIIIRNAi-enhanced TGF-*β*3-induced chondrogenic differentiation of hMSCs. Cells from T-SiNC, T-SiNC + SB, T-SiT*β*RIII, and T-SiT*β*RIII + SB groups were cultured for 14 days. (a) Alcian blue staining for GAG expression and immunohistochemistry staining for COL II expression. *n* = 3, scale bar = 50 *μ*m. (b) GAG content was quantitatively analyzed and normalized by DNA content. (c) mRNA levels of SOX9 and COL2A1 were measured by real-time PCR. Error bars represent the means ± SD, *n* = 3. ^∗^*P* < 0.05 versus T-SiNC group, ^#^*P* < 0.05 versus T-SiT*β*RIII group.

We have shown that SB431542 blocked T*β*RIII RNAi-activated TGF-*β* signaling. We next investigated whether it was sufficient to inhibit the ability of T*β*RIII RNAi-enhanced TGF-*β*3-induced chondrogenic differentiation of hMSCs. As shown in Figure 7, GAG and COL II secretion increased significantly in the T-SiT*β*RIII group (Figures 7(a) and 7(b)), as well as mRNA levels of cartilage-specific genes (SOX9 and COL2A1), as compared with that in the T-SiNC group (Figure 7(c); *P* < 0.05). Cartilage-specific protein and gene expression decreased in the T-SiNC + SB and T-SiT*β*RIII + SB groups as compared with cartilage-specific protein and gene expression in the T-SiNC and T-SiT*β*RIII groups (Figures 7(a)–7(c); *P* < 0.05).

## 4. Discussion

T*β*RIII is an abundant TGF-*β* receptor, which is present in many cell types [9, 13]. We previously demonstrated that hMSCs expressed high levels of T*β*RIII and that TGF-*β*3 reduced the expression of T*β*RIII at mRNA and protein levels [15]. Other studies demonstrated the specific role of TGF-*β*1 in decreasing T*β*RIII mRNA and protein levels in cancer cells [20, 21]. These findings were similar to those of our own study. Hempel et al. [21] showed the TGF-*β*1-mediated downregulation of T*β*RIII mRNA expression by exerting effects on the ALK5/Smad2/3 pathway of the TGF*β*R3 gene proximal promoter. Three highly homologous isoforms of TGF-*β* in humans (TGF-*β*1, TGF-*β*2, and TGF-*β*3) share a similar receptor complex and signaling pathway [20]. Therefore, the mechanism underlying the suppression by TGF-*β*3 on T*β*RIII may be the same as that involved in TGF-*β*1-mediated downregulation of T*β*RIII.

In recent years, many studies have investigated the effects of altering the expression level of T*β*RIII and its roles on mediating cell migration, invasion, growth, and differentiation in several cell types, including cancer and epithelial cells [13, 20, 22, 23]. However, the regulatory roles of T*β*RIII in MSCs have not been explored. RNAi is a powerful tool for studying protein function [24]. Lentiviral vectors encoding short hairpin RNAs (shRNAs) or microRNAs (miRNAs) can be used to specifically knock down target mRNAs [25]. Besides the ability to transfer vectors in primary and nondividing cells, the reverse-transcribed lentiviral vector is integrated in the genome, allowing stable expression of the gene or shRNA of interest [26]. We used GV248-based lentiviral vectors for delivery of shRNAs, precursors of T*β*RIII siRNA, into MSCs to suppress T*β*RIII gene expression. This vector coexpressed enhanced GFP, a reporter gene, permitting infected cells to be detected by fluorescence microscopy. Three days after infection with Lenti-siT*β*RIII or Lenti-NC, fluorescence microscopy images revealed efficient infection of MSCs with lentiviral vectors. The data also demonstrated that the lentiviral vector-based shRNA vector effectively downregulated mRNA and protein expression of T*β*RIII. These data indicated that Lenti-siT*β*RIII MSCs could be used as a powerful tool for studying the effect of the siT*β*RIII gene on MSC chondrogenesis.

Our study is the first to demonstrate that T*β*RIII knockdown in MSCs enhanced TGF-*β*3-induced chondrogenic differentiation, indicating a role for T*β*RIII as a negative modulator of MSC chondrogenesis. Previous research reported that the functional impact of T*β*RIII on TGF-*β* ligand signaling was cell-type dependent [9]. Li et al. [13] demonstrated that transfection of T*β*RIII-containing plasmid DNA dramatically promoted a TGF-*β*1-induced decrease in cell viability, apoptosis, and cell arrest. On the other hand, Eickelberg et al. indicated that the expression of T*β*RIII in renal epithelial LLC-PK1 cells resulted in inhibition of TGF signaling [27]. They also suggested that T*β*RIII with larger proteoglycans negatively modulated TGF-*β*1-induced cellular responses, whereas T*β*RIII containing smaller proteoglycans enhances TGF-*β*1-induced cellular responses [27]. These results suggested that T*β*RIII in MSCs may contain larger proteoglycans.

TGF-*β*s (TGF-*β*1, TGF-*β*2, and TGF-*β*3, resp.) are small secreted signaling proteins, each of which signals through T*β*RI and T*β*RII. In contrast to T*β*RI and T*β*RII, T*β*RIII is a membrane-anchored nonsignaling receptor, and its function is to bind and concentrate TGF-*β* isoforms on the cell surface and promote the binding of T*β*RII and recruitment of T*β*RI [11]. Chen et al. [15] demonstrated that T*β*RI was recruited to form T*β*RIII/T*β*RII/T*β*RI ternary complexes that it had two forms: complex I and complex II. Complex I contained more T*β*RII than T*β*RI, underwent clathrin-mediated endocytosis, and transduced signals in endosomes. Complex II, which contained more T*β*RI than T*β*RII, underwent caveolae/lipid-raft-mediated endocytosis and rapid degradation. The formation of these complexes was regulated by the proteoglycan moiety of T*β*RIII and altered the expression of T*β*RII or T*β*RI [15]. Herein, we demonstrated that T*β*RIII RNAi decreased T*β*RI expression and increased T*β*RII expression in the T-SiT*β*RIII group when MSCs were cultured with TGF-*β*3 as compared with the T-SiNC group (Figure 4). The data, combined with the findings of previous reports [15], indicated that T*β*RIII RNAi increased the ratio of T*β*RII to T*β*RI of the receptor complex and formed complex I, therefore enhancing TGF-*β*3 signaling in MSC chondrogenesis.

TGF-*β* signals predominantly bind to the ALK5 receptor (T*β*RI) and subsequently activate C-terminal Smad 2/3 phosphorylation. This signaling route stimulates the production of matrix components [19]. The present study also showed that T*β*RIII RNAi further enhanced the TGF-*β*3-activated downstream Smad2/3 signaling pathway (Figure 5). In addition, SB431542, a T*β*RI inhibitor, as well as dominant negative Smad2/3 specifically [28], significantly reversed T*β*RIII RNAi-mediated increases in TGF-*β*3-induced MSC chondrogenesis (Figures 6 and 7). These data indicated that T*β*RIII knockdown enhanced TGF-*β*3-induced MSC chondrogenesis via the Smad2/3 signaling pathway.

## 5. Conclusions

This study demonstrated that TGF-*β*3 inhibited the expression of T*β*RIII. T*β*RIII RNAi enhanced TGF-*β*3-induced chondrogenic differentiation of hMSCs by increasing the ratio of T*β*RII to T*β*RI in the receptor complex and further activating downstream Smad2/3 signaling. These findings contribute to the understanding of the molecular mechanisms that control chondrogenic differentiation of MSCs and provide a potential strategy (i.e., modification of MSCs by T*β*RIII knockdown for cell-based cartilage tissue engineering).

## Figures and Tables

**Table 1:** Primers used for real-time PCR.

Gene	Forward primer (5′ to 3′)	Reverse primer (5′ to 3′)
GAPDH	5′-AGAAAAACCTGCCAAATATGATGAC-3′	5′-TGGGTGTCGCTGTTGAAGTC-3′
*β*-Actin	5′-GACTTAGTTGCGTTACACCCTTTC-3′	5′-GCTGTCACCTTCACCGTTCC-3′
Col2A1	5′-GGCAATAGCAGGTTCACGTACA-3′	5′-CGATAACAGTCTTGCCCCACTT-3′
SOX 9	5′-AGCGAACGCACATCAAGAC-3′	5′-GCTGTAGTGTGGGAGGTTGAA-3′
T*β*RI	5′-ATTACCAACTGCCTTATTATGA-3′	5′-CATTACTCTCAAGGCTTCAC-3′
T*β*RII	5′-ATGGAGGCCCAGAAAGATG-3′	5′-GACTGCACCGTTGTTGTCAG-3′
T*β*RIII	5′GTGTTCCCTCCAAAGTGCAAC-3′	5′-AGCTCGATGATGTGTACTTCCT-3′

GAPDH: glyceraldehyde-3-phosphate dehydrogenase; COL2A1: collagen type II; SOX9: SRY- (sex determining region Y-) box 9; T*β*RI/II/III: recombinant human transforming growth factor-*β* receptor type I/II/III.

## Data Availability

The data used to support the findings of this study are available from the corresponding author upon request.
